# SHAPEwarp-web: sequence-agnostic search for structurally homologous RNA regions across databases of chemical probing data

**DOI:** 10.1093/nar/gkae348

**Published:** 2024-05-06

**Authors:** Niek R Scholten, Dennis Haandrikman, Joshua O Tolhuis, Edoardo Morandi, Danny Incarnato

**Affiliations:** School for Life Science and Technology, Hanze University of Applied Sciences, 9747 AS Groningen, The Netherlands; School for Life Science and Technology, Hanze University of Applied Sciences, 9747 AS Groningen, The Netherlands; School for Life Science and Technology, Hanze University of Applied Sciences, 9747 AS Groningen, The Netherlands; Department of Molecular Genetics, Groningen Biomolecular Sciences and Biotechnology Institute (GBB), University of Groningen, 9747 AG Groningen, The Netherlands; Department of Molecular Genetics, Groningen Biomolecular Sciences and Biotechnology Institute (GBB), University of Groningen, 9747 AG Groningen, The Netherlands

## Abstract

RNA molecules perform a variety of functions in cells, many of which rely on their secondary and tertiary structures. Chemical probing methods coupled with high-throughput sequencing have significantly accelerated the mapping of RNA structures, and increasingly large datasets of transcriptome-wide RNA chemical probing data are becoming available. Analogously to what has been done for decades in the protein world, this RNA structural information can be leveraged to aid the discovery of structural similarity to a known RNA (or RNA family), which, in turn, can inform about the function of transcripts. We have previously developed SHAPEwarp, a sequence-agnostic method for the search of structurally homologous RNA segments in a database of reactivity profiles derived from chemical probing experiments. In its original implementation, however, SHAPEwarp required substantial computational resources, even for moderately sized databases, as well as significant Linux command line know-how. To address these limitations, we introduce here SHAPEwarp-web, a user-friendly web interface to rapidly query large databases of RNA chemical probing data for structurally similar RNAs. Aside from featuring a completely rewritten core, which speeds up by orders of magnitude the search inside large databases, the web server hosts several high-quality chemical probing databases across multiple species. SHAPEwarp-web is available from https://shapewarp.incarnatolab.com.

## Graphical Abstract

**Graphical Abstract:**
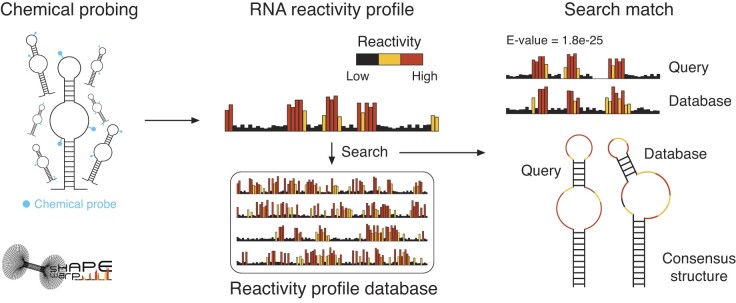

## Introduction

The ability of RNA molecules to adopt specific folds is paramount to their functions (1). In this regard, mapping RNA structures at scale is a key step toward understanding their biological roles. The characterization of RNA folding can be a tedious, time-consuming task. Of the variety of methods available to study RNA structure, chemical probing has gained substantial popularity over the last decade, particularly because its synergistic use with massive parallel sequencing enables the rapid structural interrogation of thousands of transcripts in a single experiment (2).

Identifying RNAs sharing structural homology holds importance in a variety of contexts. First, RNA molecules often exhibit shared structural motifs associated with specific biological functions. Rapid advances in the fields of genomics and transcriptomics have revealed how the majority of the nonrepetitive genome of higher metazoans is transcribed (3,4), yielding thousands of transcripts of unknown functional significance, often lacking the ability to encode for proteins. In this context, identifying structural similarity across these RNAs could aid the inference of their biological functions. Second, the evolutionary conservation of RNA structures is typically indicative of functional significance (5). Discovering RNAs with shared folding across different species can provide insights into evolutionarily conserved functions and accelerate the discovery of regulatory elements. Third, RNA structures are getting increasingly recognized as targets for small molecule drugs (6,7). Identifying structurally similar RNAs can aid in predicting off-targets and consequent side effects of RNA-targeted drugs. Additionally, understanding the structural diversity of RNA targets can guide the design of specific RNA-targeting drugs with improved efficacy and selectivity.

We have previously introduced the SHAPEwarp algorithm (8). Given an RNA reactivity profile derived from a chemical probing experiment, typically selective 2′-hydroxyl acylation analyzed by primer extension (SHAPE) (9), this method allows identifying, in a sequence-agnostic fashion, structurally homologous RNAs in a database of chemical probing-derived RNA reactivity profiles. Briefly, SHAPEwarp identifies ungapped groups of short segments (high scoring groups, or HSGs) showing highly similar reactivity profiles between a query and a target RNA. Each HSG serves as a seed from which the alignment is bidirectionally extended using a banded semi-global alignment algorithm that incorporates features of both the Gotoh’s Smith–Waterman algorithm with affine gap penalties (10) and dynamic time warping. By default the alignment solely relies on the reactivity profiles, although sequence can be optionally considered. However, configuring and running this tool requires significant computational resources and technical know-how. To simplify this task, we introduce here SHAPEwarp-web, a web server that provides the full capabilities of SHAPEwarp, via an easy and interactive user interface.

## Materials and methods

### SHAPEwarp core

**Figure 1.**
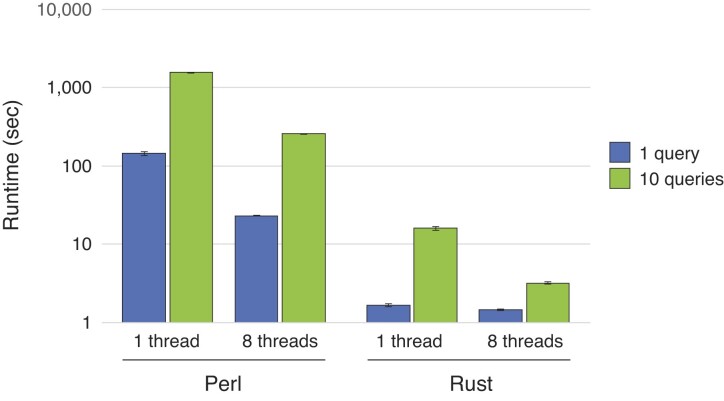
Comparison between the runtimes of the original Perl implementation of SHAPEwarp versus the new Rust implementation. The benchmark was performed by searching either 1 or 10 × 200-nucleotide-long queries against a ∼30 kb database, using either one or eight processors.

In its original implementation, the SHAPEwarp algorithm (8) was written in object-oriented Perl. In order to enable efficient search within large databases with multiple queries, the SHAPEwarp core has been entirely rewritten in Rust. This new implementation is, on average, two orders of magnitude faster (Figure 1). For reference, the Rust implementation enables searching 50 × 200-nucleotide-long queries against a database of ∼30 kb in just ∼82 s on a single thread, as compared to ∼2.9 h for the Perl implementation. The stand-alone version of SHAPEwarp is available on GitHub (https://github.com/dincarnato/SHAPEwarp). A complete manual detailing all SHAPEwarp parameters is available on Read the Docs (https://shapewarp-docs.readthedocs.io/en/latest/).

### SHAPEwarp-web

The frontend of SHAPEwarp-web is implemented in HTML5 (https://www.w3.org/TR/html5/), CSS3 and jQuery (https://jquery.com). The backend is implemented in Perl. SHAPEwarp-web is freely accessible at https://shapewarp.incarnatolab.com/. The source code of SHAPEwarp-web is available from GitHub (https://github.com/dincarnato/SHAPEwarp-web), which enables the web interface to be also deployed locally.

### Available databases

At present, SHAPEwarp-web features 13 databases: (i) 5 in-cell *Homo sapiens* transcriptome-wide icSHAPE datasets (H9 hESC, HEK293, HeLa, HepG2, K562) (11); (ii) 1 in-cell *Mus musculus* transcriptome-wide icSHAPE dataset (mES) (11); (iii) 4 *in vitro* and in-cell SARS-CoV/SARS-CoV-2 SHAPE-MaP datasets (8,12); (iv) 2 in-cell ZIKV icSHAPE datasets (Asian and African lineages) (13); and (v) 1 combined in-cell ribosomal RNA (rRNA) SHAPE-MaP dataset from multiple species (*Escherichia coli*, *Bacillus subtilis*, *Saccharomyces cerevisiae*, *H. sapiens*) (14). New databases will be added as they become publicly available.

## Results

### Job submission

**Figure 2.**
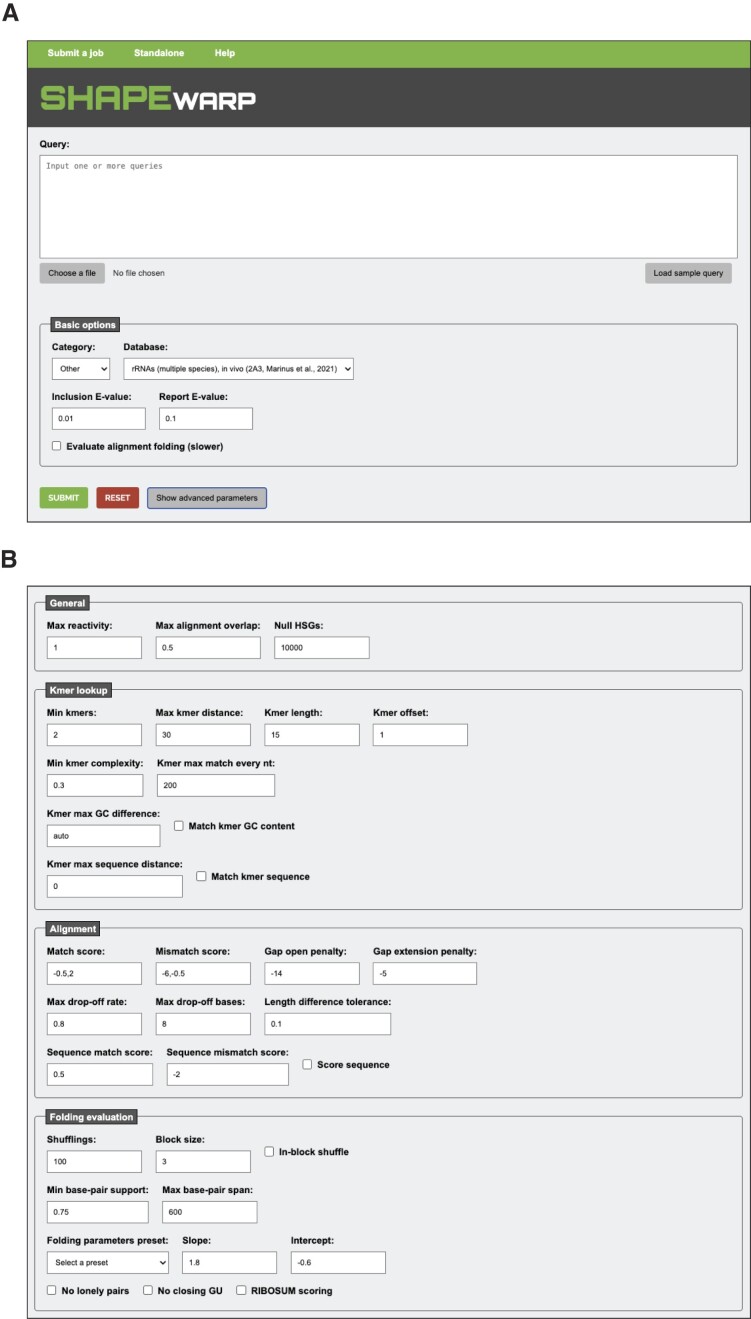
(**A**) The SHAPEwarp-web job submission page. Search queries can be directly pasted into the search text-box or uploaded as text files. Databases are grouped by category. Selecting a different entry from the ‘Category’ drop down will update the list of available databases. (**B**) Clicking the ‘Show advanced parameters’ will reveal an extensive panel of advanced search options.

Job submission requires queries to be provided in the standard SHAPEwarp format. Each query is composed of three lines: (i) query ID; (ii) sequence; and (iii) a comma-separated list of per base chemical probing reactivities. Queries can be pasted in the search text-box or provided as a text file. The current size limit for each individual query is 500 nucleotides, while the cumulative size limit is 5000 nucleotides. The basic job submission page (Figure 2A) allows selecting the database the query should be searched against, adjusting the *E*-value threshold to return database matches and enabling the evaluation of the match folding. Match folding evaluation relies upon the RNAalifold software (15) of the ViennaRNA package (16), and it allows modeling the consensus structure of the aligned query and matched database entry. Two different *E*-value thresholds can be set. The inclusion *E*-value threshold defines the maximum *E*-value to report a match as significant. The report *E*-value threshold defines the maximum *E*-value to have a match being listed in the results page. Extensive user customization of the search parameters is also possible via the advanced parameter panel (Figure 2B).

### Results page

**Figure 3.**
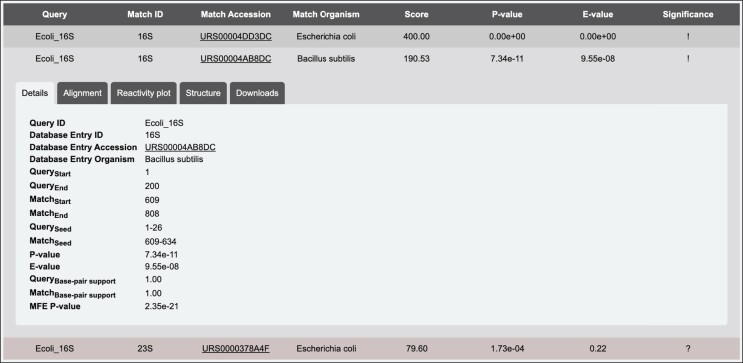
Example of a search results table. Clicking on each row will expand the detail tabs for that specific match.

Upon submission, the job is assigned a unique ID. The results page, which can be bookmarked for later retrieval, will keep automatically refreshing until the job is completed. Results will be stored on the server for up to 3 months. Results are parsed and rendered in the form of a dynamic table. Table rows are color-coded according to the significance of the match. Matches that passed both the inclusion and report *E*-value thresholds are colored in dark gray, while matches that did not pass the inclusion *E*-value but passed the report *E*-value are colored in red. Clicking on each row of the table will open a detailed view of the match, with four main tabs (Figure 3). The Details tab contains details about the match, such as start and end positions of the alignment in both the query and matched database entry, the match *E*-value, the matched organism and database transcript ID. Transcript IDs are cross-referenced to the relevant public databases, such as GenBank, RNAcentral or ENSEMBL. The Alignment tab contains the aligned sequences of query and matched database entry. The Reactivity plot tab contains an interactive bar plot of the aligned chemical probing reactivity profiles of query and matched database entry. Plots are rendered using Plotly.js (https://plotly.com/javascript/) and can be exported as SVG publication-quality graphics. The Downloads tab contains links to download the sequence alignment in Stockholm format, and to the aligned reactivity profiles in JSON format. A fifth Structure tab might be present in case the alignment folding evaluation option was enabled at job submission. This tab contains a representation of the query and database match folded according to the consensus structure inferred by RNAalifold, and with superimposed chemical probing reactivities. Secondary structure plots are rendered using R2DT (17) and downloadable as SVG graphics from the Downloads tab. If the structure is computed, this will be included in the alignment Stockholm file.

### Use cases

**Figure 4.**
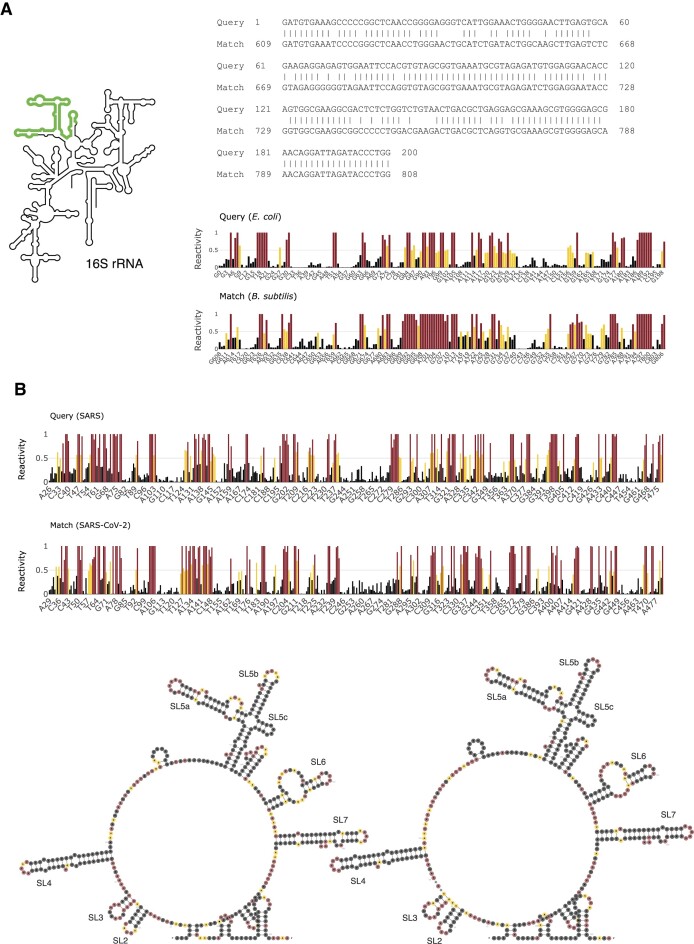
(**A**) Sequence (top) and SHAPE reactivity profile (bottom) alignments for the first use case. The query, spanning nucleotides 601–800 of the *E. coli* 16S rRNA, matched nucleotides 609–808 of the *B. subtilis* 16S rRNA. (**B**) SHAPE reactivity profile alignment (top) and consensus secondary structure inferred by RNAalifold from the SHAPEwarp alignment (bottom) for the SARS (query) and SARS-CoV-2 (database match) 5′ UTRs. Bar plots have been generated using Plotly.js. Secondary structure plots have been generated using R2DT (17).

To demonstrate the use of SHAPEwarp-web, we have selected two cases. The first case involves searching a 200-nucleotide-long segment (position: 601–800 nt) of the *E. coli* 16S rRNA, obtained by probing living *E. coli* with the 2A3 SHAPE reagent (14), in a database of rRNA SHAPE reactivity profiles from multiple species. This case scenario can be reproduced by clicking the ‘Load sample query’ button in the job submission page. Aside from matching the *E. coli* 16S rRNA itself, the search returns a second match (*E*-value = 9.27 × 10^−8^), corresponding to the structurally homologous region within the *B. subtilis* 16S rRNA (Figure 4A). The second case involves searching the reactivity profile of the first 500 nucleotides of the SARS virus genome (gRNA), obtained by *in vitro* probing of the refolded gRNA with the 2A3 SHAPE reagent, against a database generated by probing the SARS-CoV-2 gRNA under the same conditions (8). The sole significant match (*E*-value = 0) corresponds to the 5′ untranslated region (UTR) of the SARS-CoV-2 genome (Figure 4B). Enabling the folding evaluation option produces a consensus structure model that accurately recovers nearly all stem-loops of sarbecovirus 5′ UTRs (SL2 through SL7) (18). These use cases demonstrate how SHAPEwarp-web can be used to both identify structurally related RNAs and accurately model their structure.

## Concluding remarks

In conclusion, SHAPEwarp-web offers a versatile and efficient tool for identifying structurally similar RNA regions across large databases of RNA chemical probing data. By providing a user-friendly web interface and by vastly improving the computational efficiency of the search algorithm, this tool will enable researchers to rapidly explore structural features of transcriptomes. As the popularity of chemical probing increases, new high-quality datasets across different species will become available, which will further enhance its utility in uncovering conserved structural motifs and investigating the intricate relationships between RNA structure and function.

## Data Availability

SHAPEwarp-web is available from https://shapewarp.incarnatolab.com.
